# Algal MIPs, high diversity and conserved motifs

**DOI:** 10.1186/1471-2148-11-110

**Published:** 2011-04-21

**Authors:** Hanna I Anderberg, Jonas ÅH Danielson, Urban Johanson

**Affiliations:** 1 Department of Biochemistry, Center for Molecular Protein Science, Center for Chemistry and Chemical Engineering, Lund University, PO Box 124, S-221 00 Lund, Sweden

## Abstract

**Background:**

Major intrinsic proteins (MIPs) also named aquaporins form channels facilitating the passive transport of water and other small polar molecules across membranes. MIPs are particularly abundant and diverse in terrestrial plants but little is known about their evolutionary history. In an attempt to investigate the origin of the plant MIP subfamilies, genomes of chlorophyte algae, the sister group of charophyte algae and land plants, were searched for MIP encoding genes.

**Results:**

A total of 22 MIPs were identified in the nine analysed genomes and phylogenetic analyses classified them into seven subfamilies. Two of these, Plasma membrane Intrinsic Proteins (PIPs) and GlpF-like Intrinsic Proteins (GIPs), are also present in land plants and divergence dating support a common origin of these algal and land plant MIPs, predating the evolution of terrestrial plants. The subfamilies unique to algae were named MIPA to MIPE to facilitate the use of a common nomenclature for plant MIPs reflecting phylogenetically stable groups. All of the investigated genomes contained at least one *MIP *gene but only a few species encoded MIPs belonging to more than one subfamily.

**Conclusions:**

Our results suggest that at least two of the seven subfamilies found in land plants were present already in an algal ancestor. The total variation of MIPs and the number of different subfamilies in chlorophyte algae is likely to be even higher than that found in land plants. Our analyses indicate that genetic exchanges between several of the algal subfamilies have occurred. The PIP1 and PIP2 groups and the Ca^2+ ^gating appear to be specific to land plants whereas the pH gating is a more ancient characteristic shared by all PIPs. Further studies are needed to discern the function of the algal specific subfamilies MIPA-E and to fully understand the evolutionary relationship of algal and terrestrial plant MIPs.

## Background

### General function and structure of MIPs

Major Intrinsic Proteins (MIPs) are pore forming membrane proteins found in virtually all types of organisms. They have been shown to facilitate the passive transport of a wide range of small, polar molecules such as water, glycerol and urea [1-5]. MIPs are thought to have evolved through an internal gene duplication creating a direct repeat resulting in the twofold quasi symmetry of the structure [6]. Even though the overall pairwise sequence similarities can be low, all MIPs share some structural features such as having six transmembrane helices (H1-H6), connected by five loops (loop A-loop E), and two highly conserved NPA motifs. The NPA motifs are located at the N-terminal end of two half helices, HB and HE, formed by parts of loop B and loop E respectively. These two half helices are inserted from opposite sides of the membrane and meet to form one of two selectivity regions of the pore. The positive charges formed by the helical dipoles of HB and HE are focused on the NPA motifs, thereby effectively obstructing the passage of protons by means of electrostatic repulsion [7]. The second restriction site of the MIP pore is called the aromatic/arginine (ar/R) selectivity filter and consists of four amino acid residues forming the narrowest part of the pore. It is thought that the amino acid residue composition of this constriction site is the major determinant of the substrate specificity of MIPs [8].

Terrestrial plants have more isoforms and a wider variety of MIPs than any other group of organisms. Even in the genome of a relatively simple land plant like a moss 23 different MIPs, divided on seven subfamilies, are encoded [9]. Little is known on why, when and how all these subfamilies evolved in plants. Land plants are thought to descend from fresh water green algae and thus identification and studies of algal MIPs can potentially provide clues to the origin and early evolution of plant MIPs. The complete set of MIPs in nine algal genomes were therefore identified, analysed and compared to land plant MIPs in this work.

### Evolution and phylogeny of green algae

**Figure 1:**
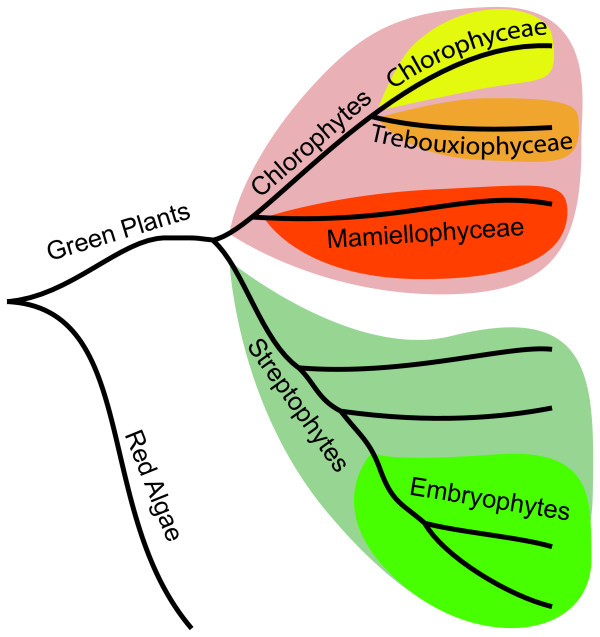
**Schematic phylogeny of green plants**. The chlorophytes (pink) and the streptophytes (light green) constitute the two phyla of green plants. The chlorophytes are further divided into a number of classes including chlorophyceae (yellow), trebouxiophyceae (orange) and mamiellophyceae (red) whereof mamiellophyceae is the basal clade. Terrestrial plants (embryophytes; green) are part of the streptophyte phylum. The position of red algae is indicated to root the schematic tree.

The clade of green plants (viridiplantae) together with the glaucophytes and the red algae (rhodophytes) form the larger monophyletic clade archaeplastida (the plant kingdom) which include all organisms with a chloroplast of primary endosymbiotic origin. The green plants are divided into the chlorophytes (consisting only of algal species) and streptophytes (containing both algae and land plants) and these clades are thought to have split 725-1200 Million Years Ago (MYA) [10-12] (Figure 1). The chlorophytes are further divided into several classes and even though the internal relationship between many of these classes is unresolved, there is a general consensus that mamiellophyceae is basal to chlorophyceae and trebouxiophyceae, which both belong to the well-defined UTC clade [13]. The other group of green plants, streptophytes, consists of a few classes of green algae (collectively known as the paraphyletic group charophyta) and the monophyletic group of land plants (embryophyta). The green algae studied in this work all belong to the chlorophyte clade with the *Ostreococcus *and *Micromonas *species belonging to the class mamiellophyceae [13], *Volvox carteri *and *Chlamydomonas reinhardtii *to the class chlorophyceae and *Coccomyxa *sp. C-169 and *Chlorella *sp. NC64A to the class trebouxiophyceae (Figure 1).

In this article, we identify 22 algal MIPs that cluster into seven subfamilies whereof two, the Plasma membrane Intrinsic Proteins (PIPs) and GlpF-like Intrinsic Proteins (GIPs), already have been characterized in land plants. All the investigated algal genomes encode at least one MIP, but only three contain more than one subfamily. The characteristics of each of the seven subfamilies are described and the evolution of the wide variety of algal MIPs, and their functions, is discussed.

## Results

### Identification and annotation of algal *MIP *genes

Nine algal genomes available at the Joint Genome Initiative (JGI) [14] were searched for encoded MIPs using protein sequences from *Physcomitrella patens *and *Chlamydomonas rheinhardtii *as queries [9,15]. This yielded 20 different *MIP *genes of which only two corresponding complete sequences and one partial sequence were also present in GenBank [16]. Less than half of the identified algal *MIP *genes had a good model in JGI [14], hence new gene models were created for the majority of the genes (Additional file 1). In one of the algal genomes (*Ostreococcus tauri*) no *MIP *genes were found initially. However, repeating the searches of all genomes using all algal MIPs discovered in the first round of searches as queries revealed two more MIP sequences, whereof one was from this species (*Ot*MIPC1;1), resulting in a total of 22 algae MIPs, see Table 1. Six of these MIPs are found in the five species belonging to the class mamiellophyceae whereas the remaining 16 MIPs are derived from the four species within the UTC clade. Coding sequences for the algae MIPs are provided in FASTA-format (Additional file 2).

**Table 1:** Identified algae MIPs

Organism	**Ver.**^**a**^	**Name**^**b**^	**GI number**^**c**^	**Gene Model**^**d**^	Comment
*Chlamydomonas reinhardtii*	4.0	*Cr*MIPD1;1	159471952	au5.g4049_t1	Previously CrMIP1
		*Cr*MIPD2;1	159466961^e^	-	Previously CrMIP2

*Volvox carteri*	1.0	*Vc*MIPD1;1		e_gw1.60.83.1	
		*Vc*MIPD2;1		-	
		*Vc*MIPD4;1^f^		fgenesh4_pg.C_scaffold_32000076	

*Coccomyxa C-169*^*g*^	2.0	*Cc*MIPA1;1		-	
		*Cc*MIPD 1;1		-	
		*Cc*MIPD3;1		-	
		*Cc*PIP4;1		-	
		*Cc*PIP4;2		-	
		*Cc*GIP1;1		-	

*Chlorella NC64A*	1.0	*Cn*MIPD1;1		IGS.gm_19_00211	
		*Cn*MIPE1;1		-	
		*Cn*MIPE1;2		-	
		*Cn*MIPE1;3		-	Gap after exon 4
		*Cn*GIP1;1		-	Gap before ex7

*Micromonas pusilla CCMP1545*	2.0	*Mp*MIPC1;1		-	

*Micromonas RCC299*	3.0	*Mr*MIPC1;1		EuGene.0900010614	

*Ostreococcus lucimarinus*	2.0	*Ol*MIPB1;1	145352492	estExt_fgenesh1_pg.C_Chr_120034	EST

*Ostreococcus RCC809*	2.0	*Or*MIPB1;1		EuGene.1100010007	
		*Or*MIPE1;1		estExt_Genewise1Plus.C_chr_30268	EST

*Ostreococcus tauri*	2.0	*Ot*MIPC1;1^f^		0400010048	

^a)^Genome version at JGI [14] used for annotation ^b)^Name used for MIP in this paper ^c)^GI number for sequence in GenBank [16] if existing ^d)^Name of gene model for MIP at JGI [14] if existing ^e)^The GI number refer to a gene model with shorter 5'-end ^f)^These two MIPs were found when rechecking the genomes for MIPs and are therefore not included in the phylogenetic analysis presented in this article. Vc_MIPD2_1 does not start with methionine ^g)^Previously classified as *Chlorella vulgaris *C-169

### Sequence alignments

**Figure 2:**
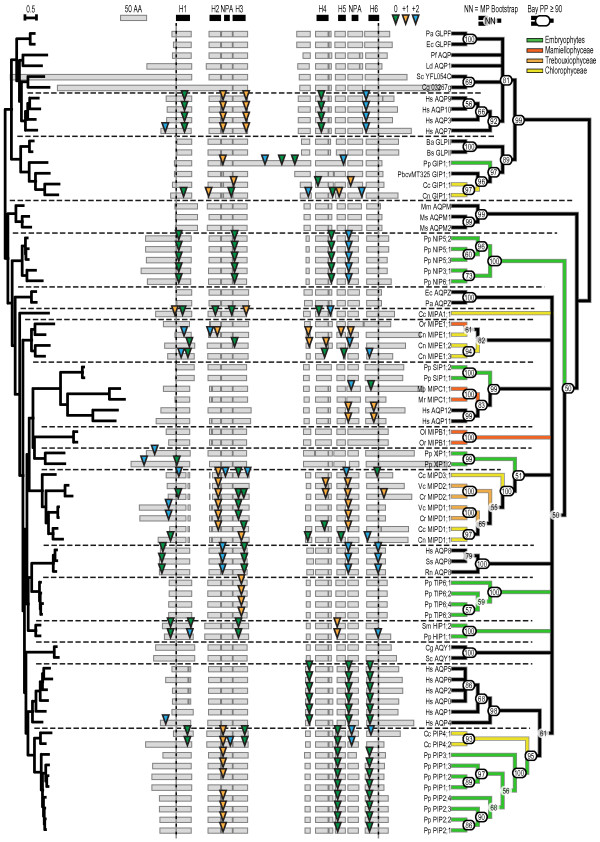
**Structural alignment, intron positions and phylogeny**. The gray boxes in the middle of the picture show the actual amino acid sequence alignment of the proteins included in the analysis whereas the black boxes at the top indicate the position of the structurally conserved elements, transmembrane helices (H1-H6) and the NPA-boxes. The central portion of the alignment used in phylogenetic analyses is delimited by the vertical dotted lines, excluding the N- and C-terminal regions. The coloured arrows indicate intron positions in the corresponding coding sequence, where the colours represent the relative position within the codon. The tree to the left is the Maximum Likelihood tree where the length of the branches is representative of sequence divergence. To the right a consensus tree based on the results from the maximum likelihood analysis is depicted, showing the stability of the clustering of sequences. The bootstrap support in percent is indicated at each node (nodes with bootstrap support lower than 50% are collapsed) and for nodes with a Bayesian posterior probability above 90% this number is also circled. Horizontal dotted lines separate clearly distinguishable clusters. The coloured branches in the consensus tree highlight from which phyla the plant MIPs were derived, see also Figures 1 and 7.

To take advantage of the high degree of structural conservation within the MIP family, a three dimensional alignment of MIP structures was constructed and used as a guide in creating a sequence alignment. Representatives of known subfamilies from all three domains of life were then added and manually aligned to the initial structure based sequence alignment. Thereafter the identified algal sequences from the first searches were added and aligned. Finally the number of reference sequences in the alignment was reduced to a more manageable subset still representing a wide variety of MIPs from bacteria, land plants, mammals and viruses. The highly divergent N- and C-terminal regions were excluded in the phylogenetic analyses and therefore no effort was put into aligning these. An overview picture of the alignment showing gaps and the positions of conserved structural elements is shown in Figure 2. Also indicated in the figure are the intron positions in the gene models and their relative position to the reading frame. Since these were not included in the dataset underlying the phylogenetic analysis, the positions can be used to verify the phylogenetic grouping of sequences. The alignment file is provided in Nexus format as Additional file 3.

### Phylogenetic analyses reveal seven groups of MIPs in algae

In order to classify the algal MIPs, their protein sequences were analysed phylogenetically together with the reference set, using both the maximum likelihood method [17] and the Bayesian method [18]. Results from these analyses are also presented in Figure 2, displaying the Maximum Likelihood tree to the left, where the branch lengths illustrate the sequence divergence, and a consensus tree to the right, summarizing the results of two different stability tests. In this tree the stability of each node is shown by the Maximum Likelihood bootstrap support value together with indications of Bayesian posterior probabilities of at least 90%.

In order to facilitate the interpretation, the phyla from which the plant MIPs are derived are indicated by colour coding of the branches in the consensus tree in Figure 2. The 22 algal MIPs cluster into seven groups whereof two are closely associated with the PIP and GIP subfamilies found in land plants [9]. These two groups of algal MIPs are therefore classified as PIPs and GIPs. Classification of the remaining five novel groups is not as straight forward and hence these groups were arbitrarily named MIPA-MIPE. There is no support for an endosymbiotic origin via the chloroplast of any these subfamilies, since no close homologs are found in cyanobacteria. In the next section the characteristics of each of the seven algal MIP groups are described more in detail.

### Algal PIPs

Two sequences from *Coccomyxa *sp. C-169 grouped basal to the PIPs of *Physcomitrella patens*, a grouping with high support from both Maximum Likelihood and Bayesian inference (Figure 2). Aside from a general overall similarity, these sequences display many of the specific features that are characteristic of PIPs, supporting a common origin and function of these proteins. The ar/R filters are almost identical to the highly conserved filter of PIPs in land plants, with the minor difference of the algal MIPs having a cysteine instead of a threonine in the third position (Table 2). In the algal sequences loop A is slightly longer (3-8 amino acids) but still contains the highly conserved cysteine found in plant PIPs [19] though offset by two amino acids. The D loop, involved in gating of land plant PIPs, has a conserved loop length and the histidine required for pH gating [20] is also present. However, the two N-terminal acidic residues indicated in calcium gating [21] are lacking. The phosphorylation site in loop B is conserved (KXSXXR) whereas the C-terminal phosphorylation site specific for PIP2s [22] is not. Furthermore, the GGGAN motif of loop C is fully conserved in one of the algal sequences but only partially in the other sequence (L**G**AS**N**). Also at the gene level there is evidence of shared ancestry for all PIPs, with two intron positions being conserved (Figure 2). Considering these specific and the general overall similarities, algal MIPs were classified as PIPs but numbered *Cc*PIP4;1 and *Cc*PIP4;2, to reflect their basal position in this subfamily.

**Table 2:** Selectivity filters and NPA-boxes

		**Ar/R selectivity filter**^**a**^				NPA motifs		
Subfamily	**Protein**^**b**^	H2	H5	**LE**_**1**_	**LE**_**2**_	Loop B	Loop E	Substrate specificity
MIPA	*Cc*MIPA1;1	H	M	M	R	NPM	NPA	
	***At*TIP2;1**	**H**	**I**	**G**	**R**	**NPA**	**NPA**	*water *[58], *urea *[59], *ammonia *[60]

MIPB	*Ol*MIPB1;1	Y	L	G	R	NPS	NAA	
	*Or*MIPB1;1	Y	F	G	R	NPS	NAA	
	**GLA**_**Llac**_	**Y**	**V**	**P**	**R**	**NPA**	**NPA**	*water, glycerol *[25]

MIPC	*Mp*MIPC1;1	L	C	G	V	NPT	NPA	
	*Mr*MIPC1;1	I	T	G	V	NPT	NPA	
	*Ot*MIPC1;1	V	M	C	P	NPV	NPS	
	***Zm*SIP2;1**^**c**^	**S**	**H**	**G**	**S**	**NPL**	**NPA**	*none *[61]
	***Os*NIP2;1**^**c**^	**G**	**S**	**G**	**R**	**NPA**	**NPA**	*silicic acid *[62], *arsenite, antimonite *[63]

MIPD	*Vc*MIPD1;1	N	A	A	R	NPA	NPA	
	*Cc*MIPD1;1	N	A	A	R	NPA	NPA	
	*Cn*MIPD1;1	N	A	A	R	NPA	NPA	
	***Cr*MIPD1;1**	**N**	**A**	**A**	**R**	**NPA**	**NPA**	*glycerol *[15]
	
	*Vc*MIPD2;1	T	L	S	R	NPA	NPA	
	*Cr*MIPD2;1	T	L	S	R	NPL	NPA	
	*Cc*MIPD3;1	S	T	A	R	NPA	NPA	
	*Vc*MIPD4;1	N	A	T	H	NPT	NPA	
	***Os*TIP4;1**	**T**	**T**	**A**	**R**	**NPA**	**NPA**	*water, glycerol *[29]

MIPE	*Or*MIPE1;1	F	H	C	R	NPA	NPA	
	*Cn*MIPE1;1	F	H	C	R	NPA	NPA	
	*Cn*MIPE1;2	A	H	C	R	NAA	NPT	
	*Cn*MIPE1;3	F	H	C	R	NPA	NPA	
	***Hs*AQP5**	**F**	**H**	**C**	**R**	**NPA**	**NPA**	*water *[34]

PIP	*Cc*PIP4;1	F	H	C	R	NPA	NPA	
	*Cc*PIP4;2	F	H	C	R	NPA	NPA	
	***At*PIP2;3**	**F**	**H**	**T**	**R**	**NPA**	**NPA**	*water *[64]

GIP	PbcvMT325GIP1;1	F	V	I	R	NPA	NPA	
	*Cc*GIP1;1	F	L	N	R	NPA	NPA	
	*Cn*GIP1;1	F	I	I	R	NPA	NPA	
	***Pp*GIP1;1**	**F**	**V**	**P**	**R**	**NPA**	**NPA**	*glycerol *[24]

^a) ^The ar/R filter is defined by four amino acid residues: one in helix 2, one in helix 5 and two in loop E ^b) ^Protein names written in bold are MIPs with studied substrate specificities used as reference. They are chosen based on the similarity of their ar/R filter to the ar/R filters of the members within the group ^c)^The MIPCs have very diverse ar/R filters, making it hard to find a reference. Therefore two MIPs with ar/R filters somewhat similar to those of the MIPCs are displayed.

### Algal GIPs

Three sequences, one from *Coccomyxa *sp. C-169 and one from *Chlorella *sp. NC64A along with a sequence from the *Chlorella *virus PbcvMT325 [AQPV1; 23] clustered together with *Pp*GIP1;1 with high support in the clade containing glycerol channels from Gram positive bacteria. The ar/R filters of these algal sequences are very similar to the one of *Pp*GIP1;1, suggesting that they are permeable for glycerol (Table 2). They also contain the among glycerol channels conserved DXXXR motif just after the second NPA box, but are missing the very long C loop unique for *Pp*GIP1;1 as well as any conserved intron positions [Figure 2; 24]. However, similar to *Pp*GIP1;1 these sequences have a shorter loop preceding the first NPA box and also atypical residues compared to other MIPs at positions T72, F89 and Q93 (EcGlpF numbering). These residues have been suggested to be involved in the packing of the core near the first NPA box [6,24]. In the light of their close association with *Pp*GIP1;1 these MIPs were named *Cc*GIP1;1, *Cn*GIP1;1 and PbcvMT325GIP1;1 respectively. It should be noted that the sequence of H4 is poorly conserved in *Cn*GIP1;1, making the functionality of this particular protein questionable.

### MIPA

The remaining five algal MIP groups were named MIPA-MIPE arbitrarily. A single sequence from *Coccomyxa *sp. C-169 showed no apparent association with any other sequence and was named *Cc*MIPA1;1. In this MIP the first NPA box is substituted to NPM, a variation that is seen at the second NPA motif in several other isoforms e.g. *Pp*NIP6;1. tBLASTn searches suggest weak similarity to plant HIPs, TIPs and animal AQP8s. In line with this the ar/R filter resembles that of *At*TIP2;1 which have been shown to be permeable to water, urea and ammonia (Table 2).

### MIPBs

Two sequences from *Ostreococcus lucimarinus *and *Ostreococcus RCC809 *form a separate group. Both MIPs have atypical NPA motifs (NPS in HB and NAA in HE) and also lack the highly conserved motif leading up to the first NPA box, suggesting an alternative conformation of this part of loop B. The ar/R filter of these sequences is similar to the one in GLA_Llac _[25] suggesting that both water and glycerol might be substrates. These sequences were named *Ol*MIPB1;1 and *Or*MIPB1;1.

### MIPCs

Three sequences from *Micromonas pusilla CCMP1545*, *Micromonas RCC299 *and *Ostreococcus tauri *grouped with the *Pp*SIPs and the human AQP11 and AQP12. These MIPs all lack a conserved glycine in H2, suggested to be important in the packing of the helices [26,27]. All three proteins have unusual substitutions in H3 and H6 where a conserved glutamine is replaced by a threonine or serine and a conserved proline changed to alanine, respectively. The C-terminal regions of the algal sequences are rich in positively charged residues, similar to those of SIPs and AQP11/12 where these residues have been suggested to have a function in ER retention [28]. Overall the ar/R filter is quite different to that of any characterised MIP and despite the fact that the conserved arginine of the ar/R filter is lacking just like in the SIPs and AQP11/12, they are not really comparable (Table 2). Even though these algal sequences firmly grouped with the *Pp*SIPs they showed an even closer association with HsAQP11 and HsAQP12. This, together with the very long branch lengths, led to the classification of these sequences as a separate subfamily and therefore named *Mp*MIPC1;1, *Mr*MIPC1;1 and *Ot*MIPC1;1 instead of being classified as SIPs.

### MIPDs

One group showed a weak association with the *Pp*XIPs. This was the most numerous group containing eight MIPs with representatives from *Coccomyxa *sp. C-169, *Chlorella *sp. NC64A, *Volvox carteri *and *Chlamydomonas reinhardtii*. Consistent with the high bootstrap value for this clade the corresponding genes share one or more conserved intron positions in all except one case. The members of this group display variation from an otherwise conserved proline in H6 and instead have glutamine, glutamate, or in one case histidine at this position. They are also distinguished by the unusually short H1 and H2 and by two strictly conserved cysteines in loop C and in the loop after HE respectively. None of these features are found in XIPs, except for a conserved cysteine in the variable C-loop that can be aligned at a corresponding position. Based upon their ar/R filter these algal sequences can be divided into two groups (Table 2). One group have filters similar to that of *Cr*MIPD1;1, which has already been shown to be permeable to glycerol [15] and one group similar to *Os*TIP4;1 [29] suggesting water and glycerol as substrate for these. The MIPs in the two groups were named MIPD1 and MIPD2 to MIPD4, respectively, in accordance with the phylogenetic classification (Figure 2; *Vc*MIPD4;1, data not shown). It should be noted that *Vc*MIPD4;1 has a unique substitution in the ar/R filter where the arginine is replaced by histidine.

### MIPEs

**Figure 3:**
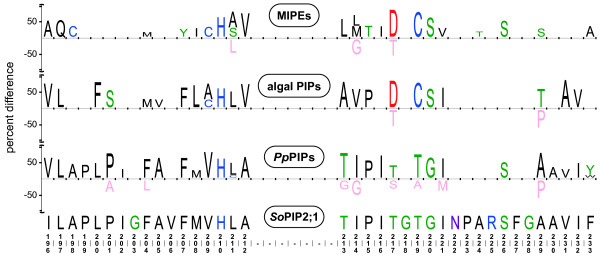
**Sequence similarities between algal PIPs and MIPEs in loop E**. Figure showing iceLogos of part of Loop E for MIPEs, algal PIPs and *P. patens *PIPs. For each of the three groups compared, the iceLogo shows the position specific over- and under representations of amino acids compared to an alignment of all MIPs included in the phylogenetic analysis. Only amino acids significantly different in the test- and reference set (P < 0.05) are shown and the size of the character reflect the difference in frequency (positive values are overrepresented whereas negative values are underrepresented in the test set). At the bottom the amino acid sequence and numbering of *So*PIP2;1 is shown to ease orientation.

**Figure 4:**
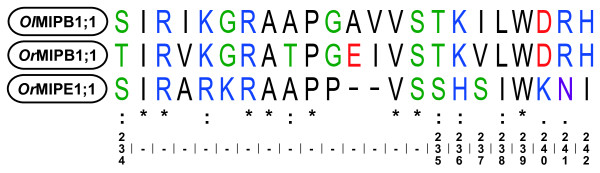
**Comparisons of the extended loop E of MIPBs and *Or*MIPE1;1**. The figure shows a sequence alignment of *Ol*MIPB1;1, *Or*MIPB1;1 and *Or*MIPE1;1. The numbering at the bottom is referring to amino acid positions in the *So*PIP2;1 sequence and the symbols over these are representing identity (*) or two degrees of similarity (: and .) of the aligned sequences.

The group containing three sequences from *Chlorella *sp. NC64A and one from *Ostreococcus *RCC809 was named MIPE. They all have ar/R filters identical to that of algal PIPs and also share a conserved motif in loop E (DGCS, where the cysteine is situated at LE_1 _of the ar/R filter) with these (Figure 3). Furthermore, phylogenetic analyses of the C-terminal region show that these parts of the MIPEs and algal PIPs are closely related (data not shown). The MIPEs also share a motif in loop C with the mammalian classical aquaporins (LXXN). In addition, *Or*MIPE1;1 shares significant sequence similarity with *Ol*MIPB1;1 and *Or*MIPB1;1 in loop E (Figure 4). It should be noted that *Cn*MIPE1;3 is missing sequence information corresponding to half of H4, loop D and most of H5 due to a gap in the available genomic sequence.

### Divergence dating

To investigate if there is any support for a horizontal gene transfer (HGT) event in the evolution of plant MIPs a divergence dating was performed. Of particular interest is the timing of the split between chlorophyte and streptophyte PIPs and the split between chlorophyte/virus and streptophyte GIPs. The PIP split was estimated to 765 MYA (geometric mean, 95% Highest Posterior Density (HPD): 543-1055 MYA) and the GIP split to 704 MYA (geometric mean, 95% HPD: 428-1054 MYA). In light of the similarity of the C-terminal part of the algal PIPs and MIPEs a divergence dating where this part was excluded from all sequences was also performed. The split of the PIPs was then estimated to 737 MYA (geometric mean, 95% HPD: 547-987 MYA). For further details see supplemented log-files (Additional files 4 and 5).

## Discussion

### Algal MIPs

Land plants contain more isoforms and subfamilies of MIPs than other organisms, but how and when this diversity evolved is presently not known. In an attempt to resolve these issues we identified and analysed all MIPs encoded in nine publicly available algal genomes. These algae belong to the chlorophyte clade, making them all equally distant relatives of land plants. At the present there are unfortunately no available genomes of the charophyte algae, which are more closely related to terrestrial plants. In total 22 MIPs were identified and divided into seven subfamilies representing a wide range of variation. Five of the subfamilies, MIPA to MIPE, are specific for algae whereas two, GIPs and PIPs, have previously only been known from terrestrial plants. In the next paragraphs the subfamilies are discussed in detail. The MIPEs are discussed together with the PIPs and MIPBs since they seem to be partially interconnected.

### Evolution of PIPs and MIPEs

The algal PIPs show many similarities with the PIPs of land plants and it is tempting to make the assumption that PIPs, with those shared features, were present already some 1000 MYA at the split of the chlorophytes and the streptophytes [10-12]. Land plant PIPs are known to be regulated by pH, Ca^2+ ^and phosphorylation and a molecular gating mechanism has been suggested. In this, several of the residues have overlapping functions in controlling the D-loop conformation in response to the different signals. However, the evolution of the gating mechanism is likely to have been a stepwise process, starting out from a primitive regulatory mechanism and then sequentially adding further functionality. The presence of an among all PIPs conserved histidine crucial for pH gating [H193 in *So*PIP2;1; 20, 21] in the algal PIPs, implies that this regulatory feature might be such a primitive mechanism. Contrary to this, the acidic amino acid residues responsible for Ca^2+ ^binding (D28 and E31 in *So*PIP2;1) are not found in the algal PIPs, suggesting that the Ca^2+^-dependent gating is a later acquired trait. This is intriguing as the proposed pH gating mechanism postulates that a salt bridge between H193 and D28 is stabilizing the closed conformation, hinting at an alternative pH dependent interaction in the algal PIPs. Such an alternative interaction might instead include a salt bridge to a phosphorylated serine located in the conserved phosphorylation motif of the B-loop (S115 in *So*PIP2;1). In the C-termini of PIP2s there is a second phosphorylation motif, also regulating the gating. The fact that this motif is only found in PIP2s and that the algal PIPs are basal to the PIPs identified in land plants, suggests that this regulation is the most recent addition to the gating mechanism.

PIPs are only present in one of the nine analysed species but an additional partial PIP sequence was found in *Parietochloris incise*, a relative to *Chlorella *NC64A (data not shown). However, their absence in all but two of the analysed species means that they have been lost not just once but at least three times in the chlorophytes if a strict vertical inheritance is assumed. An alternative explanation of the observed erratic distribution of PIPs would be a HGT between ancestors of embryophytes and the chlorophyceae, a scenario that would only require two steps.

The fact that the C-terminal part of the algal PIPs, after an among PIPs conserved intron, is more closely related to the corresponding part of the MIPEs might suggest shuffling of DNA between the different *MIP *genes (Figures 2 and 3). The ar/R filters suggest that both algal PIPs and MIPEs are water specific channels and thus functionally redundant in this aspect, which also is consistent with the finding that *MIPEs *and *PIPs *are not detected in the same genome of extant organisms. Regardless of inclusion of the MIPE-like part, the divergence dating indicates that the split between algal and embryophyte PIPs happened some 750 MYA long before the evolution of terrestrial plants, when the two lineages still might have shared an aquatic habitat [30]. The presence of a MIP in a *Chlorella *virus (PbcvMT325GIP1;1, former AQPV1) [23] suggests a possible vector for HGT. However, a vertical inheritance of PIPs cannot be excluded since the estimated divergence (547-987 MYA) overlap with the suggested time range for the split of chlorophytes and streptophytes (725-1200 MYA). Nonetheless, the dating propose that an ancestral PIP was present in the early streptophyte algal lineage leading to land plants, suggesting that extant species in the sister clade of terrestrial plants, e.g. *Chara corallina*, could encode PIPs with some of the common characteristics found in algal and embryophyte PIPs. Thus pH gating would be expected in algal PIPs but perhaps not Ca^2+ ^inhibition. In the presently known algal PIPs there is a cysteine in the ar/R filter predicting that these water channels are sensitive to inhibition by mercury [31], consistent with experimental findings in charophytes [32]. However, since this residue is in the C-terminal part of the protein, which appears to be more related to MIPEs than PIPs, the ancestral state at this position in early algal PIPs remains uncertain.

### GIPs

GIPs are found in mosses but not in seed plants. Due to the similarities between *P. patens *GIP and GLPIIs of bacteria a HGT event has been suggested. Based on sequence divergence this event is thought to have occurred about 1000 MYA [24]. A second HGT event between algae provides the simplest explanation of the sparse distribution of GIPs among plants. However, just as for the PIPs, the divergence dating of algal and *P*. *patens *GIPs is unable to discern a vertical inheritance from a scenario involving a second HGT event.

In any case, it appears that both a water channel and a glycerol uptake facilitator were acquired early on in the algal lineages leading to trebouxiophyceae and land plants.

### MIPA

There is only one unique sequence representing the proposed new subgroup of MIPA. This protein appears to be fully functional since, apart from the substitution in the first NPA box, all hallmarks of a typical MIP are present. Thus it is unlikely to be a freely evolving pseudogene and might rather be a MIP distantly related to HIPs, AQP8s or TIPs as suggested by the ar/R filter. The finding of three additional subfamilies (PIPs, GIPs, and MIPDs) in the same organism (*Coccomyxa *C-169), whereof two are also present in land plants, supports the classification of this sequence as belonging to a separate evolving subfamily rather than being a distant member, diverged by speciation from any of the other subfamilies.

### MIPBs and MIPEs

The two sequences in the MIPB subfamily are highly similar, consistent with them being derived from closely related algae. Both these proteins have unusual NPA boxes where the first and second motifs are substituted to NPS and NAA, respectively. In most other MIPs both prolines are strictly conserved indicating that they are crucial for the formation of the pore as they hold the N-terminal ends of helices HB and HE together by van der Waals interactions. The recently solved structure of PfAQP which has NLA and NPS instead of the NPA motifs suggests that the loss of van der Waals interactions between the NPA boxes in MIPBs is compensated for by formation of a hydrogen bond, from the amide nitrogen of the alanine replacing proline, to the hydroxyl group of the serine in the NPS motif [33]. The relatively rare occurrence of MIPs with alternative NPA motifs, having this type of interaction, indicates that the two kinds of interactions may not be completely functionally equivalent. Based on the interspersed distribution among the subfamilies of the alternative interaction it appears as if it has evolved independently several times. This might therefore be an example of convergent evolution resulting in similar functional characteristics. In this context it is also interesting to note that another algal MIP, *Cn*MIPE1;2, has NAA and NPT at the first and second NPA motif, respectively. In this MIP, the ar/R selectivity region deviates from the canonical water specific filter found in the other MIPEs, supporting a different transport function.

Although MIPBs are distinctly different from all other MIPs they also share some of the characteristics found in the MIPEs. As can be seen in Figure 2, the loop connecting HE and H6 is extra long in MIPBs and *Or*MIPE1;1. The high level of sequence similarity suggests that part of the *OrMIPE1;1 *gene, encoding this loop and half of HE, derive from a *MIPB *gene since none of the other MIPEs have this sequence (Figure 4).

**Figure 5:**
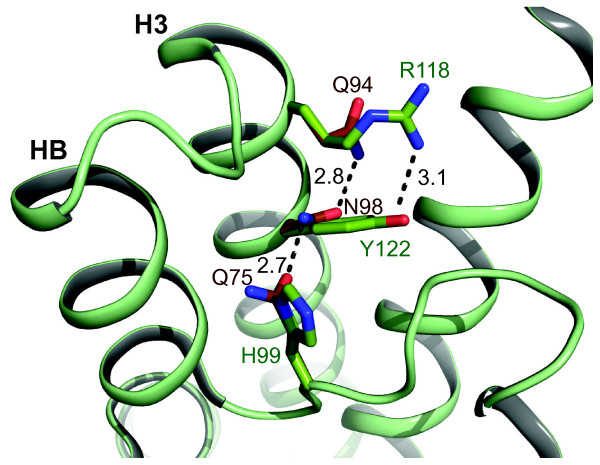
**Alternative interaction network of MIPBs and MIPEs**. MIPBs and MIPEs have unusual substitutions in helix 3 and loop B suggesting an alternative network of interactions in the packing core next to the pore at the cytoplasmic side. *So*PIP2;1 is shown in green and a model of *Ol*MIPB1;1 is superimposed in brown. The side chains at the substituted positions are drawn as sticks and their potential interactions are indicated by dashed lines with distances in Å.

In addition MIPBs and some MIPEs share unusual substitutions at positions that are part of a structurally conserved network of hydrogen bonds indirectly anchoring the short cytosolic C-terminal helix in AQP5 and the D-loop in the closed conformation of *So*PIP2;1 [34]. At two positions in this network, at the beginning of H3, all MIPBs and MIPEs have unusual substitutions where glutamine and asparagine replace arginine and tyrosine, respectively. Furthermore, an among most MIPs conserved histidine in the B loop is replaced by glutamine in MIPBs, *Or*MIPE1;1 and *Cn*MIPE1;1. These substitutions suggest that a different network of interactions can be expected in MIPBs and MIPEs (Figure 5), possibly causing changes in the properties of the cytosolic portion of the pore by affecting the conformation of the B-loop preceding the first NPA box.

### MIPCs - superaquaporins?

The MIPCs form a well-supported subfamily which associates closely with AQP11/12 and the SIPs. It might be argued that they should all be classified as one subfamily, the superaquaporins [26]. There are a few common features supporting such a classification, they are for example all rich in positively charged residues in the C-terminal, a property thought to be important for ER retention and all have a non-standard first NPA motif. However, except for these similarities no other common conserved motifs can be identified and there is a possibility that this grouping is due to long branch attraction, i.e. they are united by their dissimilarity to all other MIPs (see Figure 2), instead of by an actual shared ancestry. This might also explain why the MIPCs seem to be more closely related to AQP11/12 than the SIPs. Another more speculative explanation would be that AQP11/12 in fact originated from an algal MIPC via HGT. The uncertain evolutionary relationship of MIPCs and the other MIP groups in this clade might in fact be one of the strongest arguments for the classification of MIPCs as a separate subfamily. One unusual feature that is found in the MIPCs but not in the associated groups is the lack of the highly conserved Q and P in H3 and H6 respectively, a characteristic they share with the MIPDs (see MIPD discussion below), possibly suggesting an alternative packing of the helices in the monomer.

### MIPDs are more symmetric

**Figure 6:**
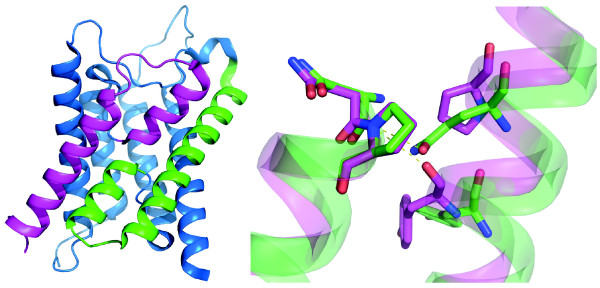
**Structural alignment of internal symmetry**. All MIPs consist of 6 transmembrane helices and two half helices, HB and HE, that together form a seventh transmembrane domain, as illustrated by the cartoon representation of the AQP4 structure to the left (PDB ID: 3GD8). Internal sequence similarities and the two-fold quasi symmetry suggest that MIPs have evolved through an internal duplication. Highlighted in green are the structural elements H3 and HB, whereas corresponding parts in the second repeat are coloured in magenta. The close up to the right depicts a structural alignment of these elements showing asparagine and proline of the NPA motif at the beginning of HB and HE as sticks. The side chain of the conserved glutamine in H3 is directed towards the nitrogen of the NPA proline in HB. In almost all MIPs the corresponding interaction in the second half of the protein is provided by a backbone oxygen in H6. This is possible due to a conserved proline hindering an α-helical H-bond within H6. Interestingly, the proline in H6 is not conserved in MIPDs which in general have glutamine or glutamate at this position, suggesting that these MIPs are more symmetrical. This structure might in fact resemble the ancestral form created by the internal duplication.

As previously mentioned all MIPs have an internal symmetry believed to derive from a duplication of an ancestral gene encoding only half of the present MIP sequence. According to this evolutionary hypothesis the first and second half of the protein were initially identical but have later diverged. Beside the NPA motifs there are symmetrically conserved residues in all corresponding transmembrane helices in the first and second half of the MIPs [35]. However, during evolution functional constraints have also selected and conserved residues that create some asymmetries in the protein. The ar/R filter is one such asymmetric feature that is now present in all MIPs. Another less studied feature is found in helices HB-H3 of the first repeat and the corresponding helices HE-H6 of the second repeat (Figure 6). In H6 there is a conserved proline preventing the formation of a hydrogen bond in the α-helix and resulting in a backbone carbonyl oxygen pointing towards the nitrogen of the proline in the NPA box. At the corresponding position in H3 there is a conserved glutamine that appears to occupy the same position as the backbone carbonyl group in H6. These features are conserved in MIP structures, but interestingly some algal MIPs display a subfamily specific variation at these two sites. MIPCs, but not SIPs or AQP11/12, have serine or threonine in H3 and alanine in H6, suggesting a different interaction in this part of the protein. Another variation is found in GIPs that have a glutamate or an asparagine in H3, conservative replacements that might not change the interactions in these areas much. However, in MIPDs the glutamine in H3 is conserved but the proline in H6 is substituted to glutamine, glutamate and in one case to histidine. This suggests that MIPDs are more symmetrical than other MIPs and thus in this regard, possibly more similar to an ancestral MIP. The fixation of the asymmetry in all other MIPs indicates a functional advantage, however the effect of this substitution is not clear and has not to our knowledge been addressed experimentally.

### Distribution and function of subfamilies

**Figure 7:**
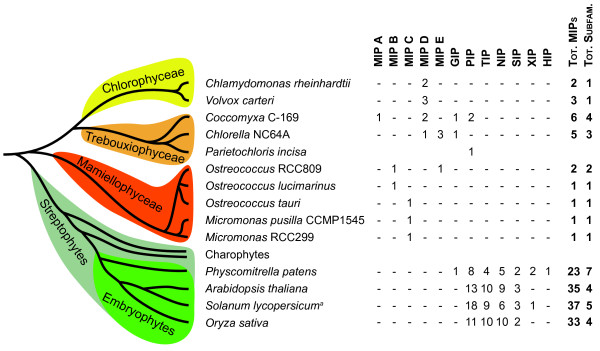
**Overview of identified MIP subfamilies in green plants**. A schematic tree showing the evolutionary relationship between green plant lineages is combined with a table summarizing the distribution of plant MIP subfamilies. MIPA-E constitutes novel subfamilies identified in this study. The PIP and the GIP subfamilies appear to have evolved before the split of the chlorophyte and the streptophyte lineages. For all plants except *S. lycopersicum *and *P. incise *the number of MIPs is derived from annotations of whole genomes [9,55,56]. ^a) ^The occurrence of MIPs in *S. lycopersicum *is based on an extensive analysis of ESTs [57].

The distribution of all the MIP subfamilies in different phylogenetic groups of plants is summarized in Figure 7. Compared to terrestrial plants, chlorophyte algal species in general have fewer subfamilies. Based on this limited dataset trebouxiophyceae have the highest number of subfamilies (3-4), followed by mamiellophyceae (1-2) whereas chlorophyceae only have a single subfamily. Still the diversity of MIPs in chlorophytes at large appears to be higher than that of land plants, resulting in a large number of subfamilies with an interspersed species distribution. A similarly complex picture was seen in a study of the ammonium transporters (AMT) presenting several chlorophyte specific subfamilies [36]. The reason for the variation is not clear but it is possible that a more careful comparison of lifestyle or habitat will reveal a logical pattern that can provide clues to the MIPs physiological function. Interestingly, trebouxiophyceae algae are not only found as free living organisms in aquatic habitats but also as symbionts in protozoa and lichen, and as a part of aeroterrestrial biofilms [37]. More specifically, some *Coccomyxa *species are free living terrestrial algae [38], whereas *Chlorella *NC64A is an endosymbiont of the ciliate *Paramecium bursaria *[39]. We speculate that the large number of MIP subfamilies found in members of trebouxiophyceae is part of an adaptation to these particular lifestyles. For an endosymbiont it is easy to envision that a facilitated exchange of solutes with the host would be beneficial, whereas the solute concentrations possible in terrestrial environments might favour a passive mode of uptake in free living non-aquatic plants.

It has been suggested that the capability to accumulate polyols, such as glycerol, is a prerequisite for algae to endure the harsh conditions in aeroterrestrial habitats [38]. In yeast, that might also experience variable conditions, it has been shown that glycerol can function as an osmoprotectant. Interestingly the opening of a glycerol facilitator, belonging to the MIP family, mediates adaptation to hypo-osmotic conditions by the rapid release of intracellular glycerol [40]. Thus, it is plausible that the physiological role of GIPs is to provide algae with the same ability to adapt to the extreme hypo-osmotic challenges in aeroterrestrial habitats posed by e.g. rain or melting snow.

Regarding the free living aquatic algae of this study we note that in some species of fish, MIPs have an important physiological function, regulating the buoyancy of the egg by adjusting the water content and thereby controlling the depth and hence the milieu for the developing egg [41,42]. It seems possible that a similar regulation can occur in free living algae in order to find optimal conditions for photosynthesis, uptake of nutrients, transport by currents or possibly to escape predators.

Studies on membrane localization and of substrate specificity will be important tools to discern the functions of the different subfamilies. At the present, we can only speculate that for example in *Coccomyxa*, MIPA could functionally correspond to TIPs in land plants and thus has a different function than the GIPs and PIPs encoded in the same alga.

As mentioned before, PIPs and MIPEs are likely to have the same or very similar functions and that might be an explanation to why they have not been found in the same organism.

MIPB and MIPC are only found in mamiellophyceae and MIPD only in chlorophyceae and trebouxiophyceae. This might indicate that these MIPs have a shared ancestry and subfamilies only reflect the evolutionary distance between the different species. Although, the phylogenetic tree presented in Figure 2 is consistent with an orthologous relationship between these algal MIPs, there is no significant support for the nodes connecting these three subfamilies. Furthermore, the ar/R selectivity filters are distinctly different, suggesting differences in substrate specificity and hence physiological function. For the plant MIPs shared between different phyla, our current understanding suggests several HGT events in the evolution of MIPs as the simplest explanation for the observed distribution and complex MIP families found in some algae and land plants. However, this could easily change as more algal genomes become available. Genomes of charophyte algae will be especially informative, bridging the gap between chlorophyte algae and land plants to give a clearer picture of the evolution of the plant MIPs.

## Conclusions

In this article the first extensive identification and classification of algal MIPs is presented. 22 different MIPs from nine species are analysed and classified into seven distinct subfamilies, representing the wide variation of MIPs found in green algae. None of the analysed species lack MIPs completely but most of them only contain a single subfamily. The multitude of subfamilies found is less likely to have been present in a common ancestor of green algae, but rather appears to have evolved later in the different algal lineages by a combination of vertical inheritance, HGT, and recombination between MIP genes. In this work, algal members of two of the seven subfamilies present in land plants are identified for the first time, suggesting that these subfamilies had formed long before land plants appeared. We also suggest that concentration gradients posed by terrestrial habitats favoured the accumulation of the many variants of passive transporters, i.e. MIPs, found in extant land living algae and embryophytes.

## Methods

### Identification and annotation of algal MIP genes

The genomes of the algae *Chlamydomonas reinhardtii*, *Volvox carteri*, *Coccomyxa *sp. C-169, *Chlorella *NC64A, *Micromonas pusilla *CCMP1545, *Micromonas *RCC299, *Ostreococcus lucimarinus*, *Ostreococcus *RCC809 and *Ostreococcus tauri*, available at the Joint Genome Initiative [14] were searched for MIP encoding genes using tBLASTn. First the *Coccomyxa *genome was searched using amino acid sequences of MIPs from *Physcomitrella patens *and *Chlamydomonas rheinhardtii *as queries. The MIPs identified in this search were then also included as queries in tBLASTn searches of the other genomes.

Genome sequences around hits were inspected for existing gene models, which were evaluated and kept if found to accurately represent a *MIP *gene. If models were missing or likely to be incorrect, new models were made by manual annotations of the genomic sequences. When satisfactory models neither existed nor could be built, hits were believed to be pseudogenes and were excluded from the analysis. Gene models were evaluated by comparing their amino acid sequences to these of known MIPs, emphasizing the existence of conserved residues and constraints on the lengths of transmembrane and loop regions.

### Sequence alignments

Initially a structural alignment was done using DeepView/Swiss-PdbViewer v4.0.1 and the structures of *Bt*AQP1 (1J4N [43]), *Ec*GLPF (1LDA, [44]), *Ec*AQPZ (1RC2, [45]), *So*PIP2;1 (1Z98, [21]), *Rn*AQP4 (2D57, [46]), *Mm*AQPM (2F2B, [47]), *Pf*AQP (3C02, [33]), *Hs*AQP5 (3D9S, [34]) and *Bt*AQP0 (2B6P, [48]). This structural alignment was then used as reference when building the alignment used for phylogenetic analyses in MEGA4 [49]. The sequence alignment used for the phylogenetic analysis was constructed by reducing the number reference sequences while trying to maintain the diversity, and by removing the highly variable N- and C-terminal regions of the sequences. The GI-numbers and amino acid sequence alignment (Nexus format) are provided as Additional files 6 and 3.

### Phylogenetic analyses

Maximum Likelihood analysis was carried out using the software PHYML (Version 2.4.4) [17]. In the analysis the JTT amino acid substitution model was used, the proportion of invariable sites was set to be estimated, the number of substitution rate categories was set to 4 and the gamma distribution parameter was set to be estimated. To assess the robustness of the best tree bootstrapping with 1000 replicates was performed. For the remaining parameters the default settings were used.

For Bayesian Inference analysis, the program MrBayes (Version 3.1.2) was used [18,50]. The analysis was run with default setting with the following changes: (1) In the likelihood model used, a portion of the sites were invariable while the rate variation of other sites were assumed to be gamma distributed, (2) The rate matrix for amino acid substitution was set to "mixed model", in which the Markov chain samples each model according to its probability, (3) The analysis was set to include 5 chains per run with a temperature of 0.2 and for each chain to run for 2 000 000 generations, sampling every 100th tree. The first 25% of the sampled trees were discarded as burn in for the analysis.

### IceLogo

IceLogos for positions 196-233 (*So*PIP2;1 numbering) of the MIPEs, the algal PIPs and for *P. patens *PIPs were created in the iceLogo program (Version 1.2) [51] using the sampling mode. Corresponding positions from all sequences of the sequence alignment were used as the reference set. The sampling type was set to terminal with the terminal index 1 (N-terminus) and the iteration size to 500.

### Divergence dating

Divergence dating was performed using the BEAST software package under a relaxed molecular clock [52]. The age of nine nodes was constrained by fossil evidence with prior probabilities distributed lognormally between a strict lower bound and a "soft" higher bound set as the 95% confidence interval. The protostomia/deuterostomia split was set to 531.5-551.9 MYA, the actinoptergyii/sarcopterygii split was set to 416.0-421.8 MYA, the archosauria/mammalian split to 312.3-330.5 MYA [53] and the bryophyte/polysporangiophyta split was set to 443.0-490.2 MYA [54]. The alignments used were similar to the one used for the phylogenetic analyses but some sequences were added to encompass all of the calibration points and some were removed to reduce complexity. The analysis was based on amino acid sequences. For further details concerning in-data and settings consult the XML-files (Additional files 7 and 8).

## Authors' contributions

JÅHD and UJ conceived and designed the study, JÅHD collected and prepared the primary dataset, HIA did all divergence dating, JÅHD and HIA contributed equally to the phylogenetical analyses. All authors participated in drafting and writing of the final manuscript.

## Supplementary Material

Additional file 1 Table S1: Gene models for algae *MIPs *with introns Click here for file

Additional file 2 FASTA-file with coding sequences of algal *MIPs* Click here for file

Additional file 3 The alignment file for Figure 2 in Nexus format Click here for file

Additional file 4 Divergence dating log-file Full length Click here for file

Additional file 5 Divergence dating log-file N-terminal Click here for file

Additional file 6 Table S2: MIPs GI numbers Click here for file

Additional file 7 Divergence dating XLM-file Full length Click here for file

Additional file 8 Divergence dating XLM-file N-terminal Click here for file
