# Supplementary material for: Algal MIPs, high diversity and conserved motifs
Source: BMC Evol Biol. 2011 Apr 21;11:110. doi: 10.1186/1471-2148-11-110 (PMC3111385; doi:10.1186/1471-2148-11-110)
Supplement: Additional file 1 — Table S1: Gene models for algae MIPs with introns [file 1471-2148-11-110-S1.DOC]

**Table S**1 Gene models for algae MIPs with introns

| **Organism** | **Ver.^a^** | **Name^b^** | **Chr/Sc^c^** | **Str.^d^** | **Start pos^e^** | **E1^f^** | **I1^f^** | **E2^f^** | **I2^f^** | **E3^f^** | **I3^f^** | **E4^f^** | **I4^f^** | **E5^f^** | **I5^f^** | **E6^f^** | **I6^f^** | **E7^f^** | **I7^f^** | **E8^f^** |
| --- | --- | --- | --- | --- | --- | --- | --- | --- | --- | --- | --- | --- | --- | --- | --- | --- | --- | --- | --- | --- |
| *Chlamydomonas* *reinhardtii* | 4.0 | *Cr*MIPD1;1 | Chr 12 | - | 7735549 | 179 | 92 | 170 | 520 | 113 | 220 | 253 | 382 | 188 |  |  |  |  |  |  |
|  |  | *Cr*MIPD2;1 | chr 17 | + | 2004305 | 60 | 455 | 100 | 304 | 113 | 350 | 33 | 230 | 124 | 238 | 90 | 325 | 174 | 241 | 179 |
| *Volvox* *carteri* | 1.0 | *Vc*MIPD1;1 | sc 60 | - | 266067 | 182 | 68 | 170 | 122 | 113 | 211 | 253 | 114 | 188 |  |  |  |  |  |  |
|  |  | *Vc*MIPD2;1 | sc55 | + | 864183 | 166 | 470 | 270 | 410 | 90 | 546 | 308 |  |  |  |  |  |  |  |  |
|  |  | *Vc*MIPD4;1^g^ | Sc32 | - | 673711 | 19 | 466 | 688 | 420 | 28 | 235 | 78 | 99 | 264 | 73 | 312 |  |  |  |  |
| *Coccomyxa* C-169^h^ | 2.0 | *Cc*MIPA1;1 | Sc 22 | - | 383728 | 118 | 389 | 47 | 207 | 102 | 248 | 96 | 386 | 85 | 221 | 65 | 143 | 71 | 288 | 304 |
|  |  | *Cc*MIPD 1;1 | Sc 2 | - | 3777044 | 157 | 351 | 146 | 716 | 126 | 564 | 91 | 577 | 329 |  |  |  |  |  |  |
|  |  | *Cc*MIPD3;1 | sc 19 | - | 1016767 | 77 | 218 | 95 | 285 | 40 | 112 | 73 | 112 | 59 | 212 | 198 | 186 | 145 | 302 | 102 |
|  |  | *Cc*PIP4;1 | Sc 9 | - | 45712 | 84 | 198 | 133 | 212 | 128 | 102 | 168 | 111 | 74 | 165 | 69 | 159 | 184 |  |  |
|  |  | *Cc*PIP4;2 | Sc. 1 | - | 2736948 | 258 | 411 | 124 | 190 | 46 | 145 | 82 | 382 | 168 | 303 | 74 | 320 | 154 |  |  |
|  |  | *Cc*GIP1;1 | Sc 17 | + | 1091287 | 262 | 237 | 169 | 1895 | 179 | 428 | 191 |  |  |  |  |  |  |  |  |
| *Chlorella* NC64A | 1.0 | *Cn*MIPD1;1 | sc 19 | - | 794166 | 75 | 116 | 286 | 174 | 62 | 167 | 147 | 199 | 104 | 130 | 256 |  |  |  |  |
|  |  | *Cn*MIPE1;1 | sc22 | + | 460068 | 56 | 243 | 54 | 517 | 44 | 174 | 245 | 165 | 88 | 174 | 45 | 131 | 389 |  |  |
|  |  | *Cn*MIPE1;2 | sc 32 | - | 365184 | 150 | 829 | 192 | 130 | 106 | 103 | 72 | 128 | 103 | 103 | 301 |  |  |  |  |
|  |  | *Cn*MIPE1;3 | sc9 | - | 1102665 | 83 | 227 | 40 | 287 | 360 | 298 | 95 | 154 | 148 |  |  |  |  |  |  |
|  |  | *Cn*GIP1;1 | sc20 | + | 938309 | 78 | 291 | 54 | 170 | 132 | 160 | 149 | 256 | 121 | 228 | 25 | 221 | 127 | 168 | 208 |

^a)^Genome version at JGI used for annotation ^b)^Name used for MIP in this paper ^c)^Chromosome or scafold location ^d)^Encoded on the postitive (+) or the negative (-) strand ^e)^Nucleotide position for start codon ^f)^Length of each exon (E) and intron (I)
