# Supplementary material for: Algal MIPs, high diversity and conserved motifs
Source: BMC Evol Biol. 2011 Apr 21;11:110. doi: 10.1186/1471-2148-11-110 (PMC3111385; doi:10.1186/1471-2148-11-110)
Supplement: Additional file 6 — Table S2: MIPs GI numbers [file 1471-2148-11-110-S6.DOC]

**Table S2** MIPs GI numbers

| **Name** | **Organism** | **GI number** | **locus tag** |
| --- | --- | --- | --- |
| *Pa*GLPF | *Pseudomonas aeruginosa* | 115583796 | PA14_17980 |
| *Ec*GLPF | *Escherichia coli* | 215263233 | E2348_C_4231 |
| *Pf*AQP | *Plasmodium falciparum* | 254832581 | PF11_0338 |
| *Ld*AQP1 | *Leishmania donovani* | 148533556 | - |
| *Sc*YFL054C | *Saccharomyces cerevisiae* | 285811827 | YFL054C |
| *Cg*03267g | *Candida glabrata* | 49524492 | CAGL0C03267g |
| *Hs*AQP9 | *Homo sapiens* | 157266306 | - |
| *Hs*AQP10 | *Homo sapiens* | 22538419 | - |
| *Hs*AQP3 | *Homo sapiens* | 22165421 | - |
| *Hs*AQP7 | *Homo sapiens* | 4502186 | - |
| *Ba*GLPII | *Bacillus anthracis* | 50082967 | GBAA_1025 |
| *Bs*GLPII | *Bacillus subtilis* | 225184640 | BSU09280 |
| *Pp*GIP1;1 | *Physcomitrella patens* | - | - |
| PbcvMT325GIP1;1 | *Paramecium bursaria chlorella virus MT325* | 94323212 | MT325_M030R |
| *Cc*GIP1;1 |  | - | - |
| *Cn*GIP1;1 |  | - | - |
| *Mm*AQPM | *Methanothermobacter marburgensis* | 12957201^a^ | 2 NA missmatch |
| *Ms*AQPM1 | *Methanosphaera stadtmanae* | 84372150 | Msp_0998 |
| *Ms*AQPM2 | *Methanosphaera stadtmanae* | 84372150 | Msp_1240 |
| *Pp*NIP5;2 | *Physcomitrella patens* | - | - |
| *Pp*NIP5;1 | *Physcomitrella patens* | - | - |
| *Pp*NIP5;3 | *Physcomitrella patens* | - | - |
| *Pp*NIP3;1 | *Physcomitrella patens* | - | - |
| *Pp*NIP6;1 | *Physcomitrella patens* | - | - |
| *Ec*AQPZ | *Escherichia coli* | 215263233 | E2348_C_0872 |
| *Pa*AQPZ | *Pseudomonas aeruginosa* | 115583796 | PA14_11660 |
| *Cc*MIPA1;1 |  | - | - |
| *Ol*MIPB1;1 |  | - | - |
| *Or*MIPB1;1 |  | - | - |
| *Pp*XIP1;1 | *Physcomitrella patens* | - | - |
| *Pp*XIP1;2 | *Physcomitrella patens* | - | - |
| *Pp*SIP1;2 | *Physcomitrella patens* | - | - |
| *Pp*SIP1;1 | *Physcomitrella patens* | - | - |
| *Mp*MIPC1;1 |  | - | - |
| *Mr*MIPC1;1 |  | - | - |
| *Hs*AQP12 | *Homo sapiens* | 156447036 | - |
| *Hs*AQP11 | *Homo sapiens* | 27370564 | - |
| *Cc*MIPD3;1 |  | - | - |
| *Vc*MIPD2;1 |  | - | - |
| *Cr*MIPD2;1 |  | - | - |
| *Vc*MIPD1;1 |  | - | - |
| *Cr*MIPD1;1 |  | - | - |
| *Cc*MIPD1;1 |  | - | - |
| *Cn*MIPD1;1 |  | - | - |
| *Hs*AQP8 | *Homo sapiens* | 45446751 | - |
| *Ss*AQP8 | *Sus scrofa* | 159461726 | - |
| *Rn*AQP8 | *Rattus norvegicus* | 2358276 | - |
| *Cg*AQY1 | *Candida glabrata* | 49524724 | CAGL0D00154g |
| *Sc*AQY1 | *Saccharomyces cerevisiae* | 285815262 | YPR192W |
| *Pp*TIP6;1 | *Physcomitrella patens* | - | - |
| *Pp*TIP6;2 | *Physcomitrella patens* | - | - |
| *Pp*TIP6;4 | *Physcomitrella patens* | - | - |
| *Pp*TIP6;3 | *Physcomitrella patens* | - | - |
| *Sm*HIP1;2 | *Selaginella moellendorffii* | - | - |
| *Pp*HIP1;1 | *Physcomitrella patens* | - | - |
| *Or*MIPE1;1 |  | - | - |
| *Cn*MIPE1;1 |  | - | - |
| *Cn*MIPE1;2 |  | - | - |
| *Cn*MIPE1;3 |  | - | - |
| *Hs*AQP5 | *Homo sapiens* | 186910293 | - |
| *Hs*AQP6 | *Homo sapiens* | 86792454 | - |
| *Hs*AQP2 | *Homo sapiens* | 209180415 | - |
| *Hs*AQP0 | *Homo sapiens* | 76253685 | - |
| *Hs*AQP1 | *Homo sapiens* | 37694061 | - |
| *Hs*AQP4 | *Homo sapiens* | 50659061 | - |
| *Cc*PIP4;1 |  | - | - |
| *Cc*PIP4;2 |  | - | - |
| *Pp*PIP3;1 | *Physcomitrella patens* | - | - |
| *Pp*PIP2;2 | *Physcomitrella patens* | - | - |
| *Pp*PIP2;3 | *Physcomitrella patens* | - | - |
| *Pp*PIP2;1 | *Physcomitrella patens* | - | - |
| *Pp*PIP2;4 | *Physcomitrella patens* | - | - |
| *Pp*PIP1;1 | *Physcomitrella patens* | - | - |
| *Pp*PIP1;2 | *Physcomitrella patens* | - | - |
| *Pp*PIP1;3 | *Physcomitrella patens* | - | - |

^a)^The sequence used in this article is the one present in the paper by Lee et al.. This differs from the sequence referred to by the GI number by 2 nucleotides.
